# Supplementary material for: Novel Driver Strength Index highlights important cancer genes in TCGA PanCanAtlas patients
Source: PeerJ. 2022 Aug 11;10:e13860. doi: 10.7717/peerj.13860 (PMC9375969; doi:10.7717/peerj.13860)
Supplement: Supplemental Information 2 [file peerj-10-13860-s002.zip › COHORTS/genes plots/detailed/2021_8_16_14_9_distribution_events_detailed_males_28.pdf]

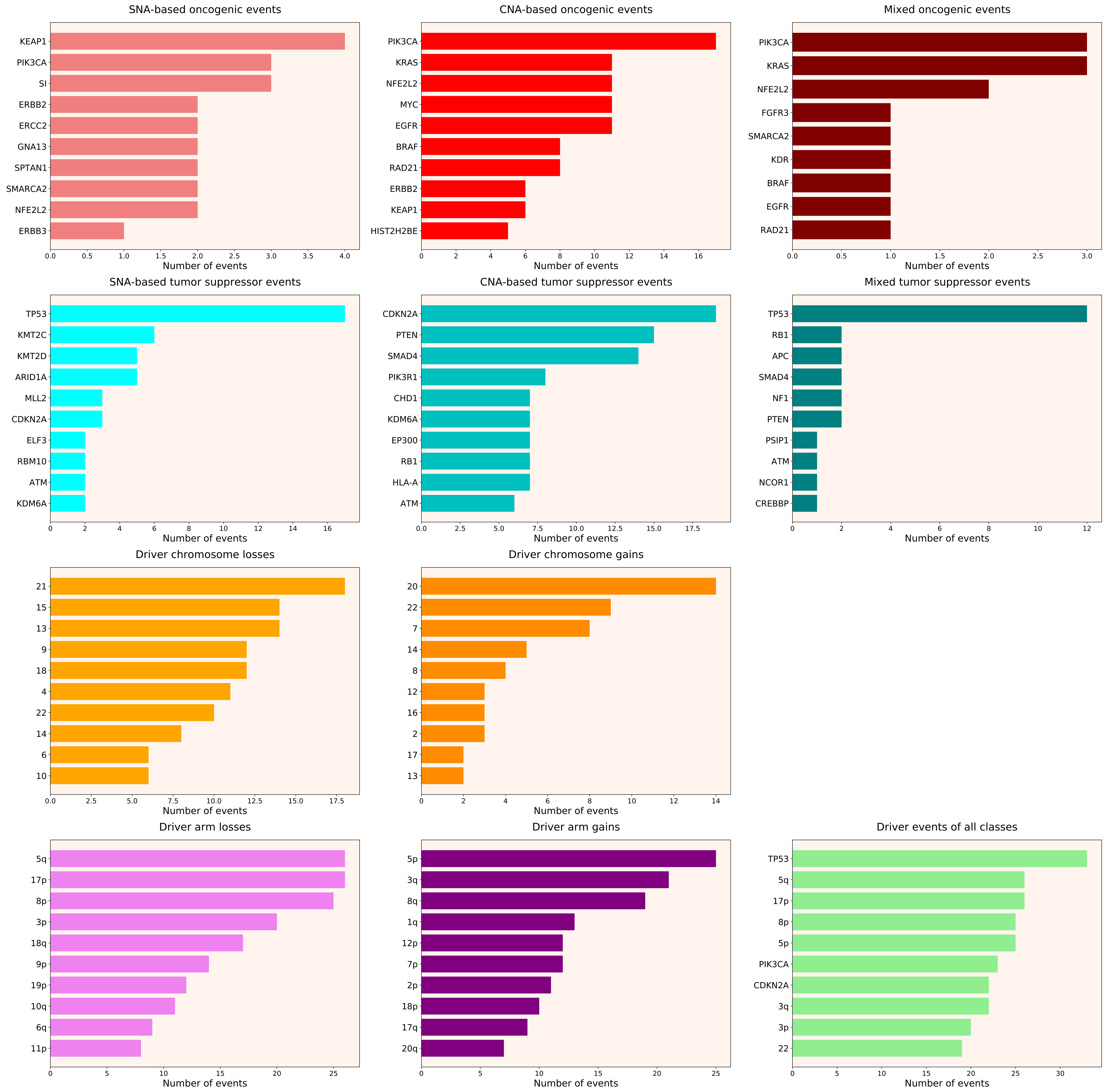
SNA-based oncogenic events
KEAP1
PIK3CA
SI
ERBB2
ERCC2
GNA13
SPTAN1
SMARCA2
NFE2L2
ERBB3
Number of events
CNA-based oncogenic events
PIK3CA
KRAS
NFE2L2
MYC
EGFR
BRAF
RAD21
ERBB2
KEAP1
HIST2H2BE
Number of events
Mixed oncogenic events
PIK3CA
KRAS
NFE2L2
FGFR3
SMARCA2
KDR
BRAF
EGFR
RAD21
Number of events
SNA-based tumor suppressor events
TP53
KMT2C
KMT2D
ARID1A
MLL2
CDKN2A
ELF3
RBM10
ATM
KDM6A
Number of events
CNA-based tumor suppressor events
CDKN2A
PTEN
SMAD4
PIK3R1
CHD1
KDM6A
EP300
RB1
HLA-A
ATM
Number of events
Mixed tumor suppressor events
TP53
RB1
APC
SMAD4
NF1
PTEN
PSIP1
ATM
NCOR1
CREBBP
Number of events
Driver chromosome losses
21
15
13
9
18
4
22
14
6
10
Number of events
Driver chromosome gains
20
22
7
14
8
12
16
2
17
13
Number of events
Driver arm losses
5q
17p
8p
3p
18q
9p
19p
10q
6q
11p
Number of events
Driver arm gains
5p
3q
8q
1q
12p
7p
2p
18p
17q
20q
Number of events
Driver events of all classes
TP53
5q
17p
8p
5p
PIK3CA
CDKN2A
3q
3p
22
Number of events
